# Assessment of Severity of Dengue Fever by Deranged Alanine Aminotransferase Levels

**DOI:** 10.7759/cureus.10539

**Published:** 2020-09-19

**Authors:** Fatima Ayaz, Muhammad Furrukh

**Affiliations:** 1 Department of Medicine, Holy Family Hospital, Rawalpindi, PAK

**Keywords:** dhf, dss, alt, dengue fever, alanine aminotransferase, dengue hemorrhagic fever, dengue shock syndrome

## Abstract

Introduction

Dengue fever (DF) is the most common arbovirus-related infection worldwide. Most of the dengue infections are asymptomatic. The clinical presentation of DF varies from mild febrile illness to dengue hemorrhagic fever (DHF) and dengue shock syndrome (DSS). Both DHF and DSS are severe forms of illness and carry higher rates of mortality. Various clinical parameters are associated with severe DF. The objective of this study was to determine any relation between elevated alanine aminotransferase (ALT) levels at presentation and development of severe DF.

Methods

This cross-sectional study was carried out at a large tertiary care hospital in Rawalpindi, Pakistan. Over a period of six months, 65 dengue patients were enrolled and their ALT levels were recorded at presentation. All the patients were managed as per guidelines in a similar way, and clinical course was followed for the development of severe forms of DF, such as DHF and DSS.

Results

Out of total 65 patients, 45 (69.2%) were males and 20 (30.8%) were females. Classical DF was present in 18 (27.7%) patients, whereas 47 (72.3%) patients developed DHF or DSS. Patients with DF had a mean ALT level of 131.67 (±244.48) U/L at presentation, whereas patients with DHF and DSS had a mean ALT level of 228.15 (±467.88) U/L at presentation. The Mann-Whitney U test was applied to compare differences between ALT levels of both groups, and p value was found to be 0.018 (<0.05). Thus, it was concluded that there was a statistical difference between ALT levels of patients with simple DF and patients with DHF or DSS.

Conclusion

Higher serum levels of ALT at presentation can give a clue about possibility of progression to severe forms of DF (DHF and DSS). Therefore, patients with higher ALT at presentation should be prioritized and monitored more rigorously.

## Introduction

Dengue fever (DF) is the most common arbovirus borne infection worldwide, and its incidence is increasing continuously throughout the globe [1]. Approximately, 50-100 million cases occur annually with around 10,000 dengue deaths per annum [2].

Most of the dengue infections are asymptomatic. The clinical presentation of DF varies from mild febrile illness to dengue hemorrhagic fever (DHF) and dengue shock syndrome (DSS) [3]. The classical DF is mild, febrile illness, while on the other hand DHF is characterized by high-grade fever, increased vascular permeability, plasma leakage, and hemorrhagic manifestations which if accompanied by shock is termed as DSS. Both DHF and DSS are severe forms of illness and carry higher rates of mortality [4]. Various clinical parameters and laboratory investigations, such as leukocyte count, prothrombin time, activated partial thromboplastin time, alanine aminotransferase (ALT) levels, aspartate aminotransferase levels, and hematocrit levels, have been studied internationally to forecast severity of infection [3,5]. Liver is not the major target organ of dengue virus, but hepatic involvement can occur due to a direct viral effect on hepatic cells or due to dysregulated immune response of the body [6,7].

In past, many national and international studies have been done to evaluate the role of liver function tests in forecasting the severity of DF. However, there was a paucity of data, pertaining specifically to relationship between elevated ALT levels and development of DHF and DSS. Early identification of DHF and DSS patients can be very crucial in resource-limited setups as these patients can be saved by strict monitoring and early aggressive management. ALT levels being widely available and inexpensive can be very useful in such less resourceful setups. Therefore, this study was designed with an objective to assess the utility of ALT as a marker of severe DF.

## Materials and methods

This cross-sectional study was carried out at the Department of Infectious Diseases of Holy Family Hospital, Rawalpindi. The duration of the study was six months (from October 2019 to March 2020).

Inclusion and exclusion criteria

Adult patients with confirmed DF as per definition of Dengue Expert Advisory Group, Punjab, Pakistan (DEAG) were included in the study [8]. However, patients with known history of chronic liver disease and patients with history of known hepatotoxic drug use within last six months were excluded from the study.

Operational definitions

DF was defined as fever of 2-12 days with at least two of the following: headache, retro-orbital pain, arthralgias/backache, myalgias, abdominal pain, rash, bleeding, and decreased urine output along with leukopenia (total leukocyte count of less than 4,000/microliter) or thrombocytopenia (platelet count less than 100,000/microliter) and either non-structural protein 1 (NS1) or dengue IgM positive. DF without any evidence of plasma leakage or shock was called as classical DF. DHF was defined as confirm DF with evidence of plasma leakage as indicated by more than 20% increase or decreases in hematocrit from baseline or radiological evidence of plasma leakage in the form of ascites, pleural effusion, increased gallbladder wall thickness, or pericholecystic fluid. DSS was defined as DF with any sign of shock like hypotension (blood pressure less than 90/60 mmHg, pulse pressure <20 mmHg, urine output <0.5 ml/Kg/hr, or capillary refill time >2 seconds).

Ethical approval

Ethical approval was obtained from the Research and Ethical Committee of Rawalpindi Medical University, Pakistan (Holy Family Hospital is allied with Rawalpindi Medical University). Reference number of approval letter is R-60/RMU.

Sampling

Sample size was calculated using the MedCalc statistical software version 19.3.1 (MedCalc Software bv, Ostend, Belgium). The minimum sample size calculated was 20 (10 in each group), using reference data from the work of Chhina et al. [9]. After informed consent, 65 patients were included in the study via non-probability convenience sampling.

Data collection and analysis

Patients were divided into two groups: group 1 included patients who had classical DF, whereas group 2 included patients with DHF and DSS. All the relevant data were noted, including age, gender, ALT levels at presentation, bleeding manifestations, and development of DHF and DSS over the course of hospital stay. Complete data were entered and then analyzed using IBM SPSS Statistics for Windows, Version 25.0 (Armonk, NY: IBM Corp). Means and standard deviations were calculated for quantitative data, such as age and hospital stay. Frequencies and percentages were computed for qualitative data, such as gender and DF/DHF/DSS. The Mann-Whitney U test was applied to compare mean ALT levels at presentation of both the groups. Null hypothesis formulated for testing stated that mean ALT levels of both the groups were equal. Confidence level was 95%, and p value of less than 0.05 was taken as significant.

## Results

Out of total 65 patients, 45 (69.2%) were males and 20 (30.8%) were females. The mean age of the patients was 36.86 (±13.9) years. Classical DF was present in 18 (27.7%) patients, whereas 41 (63.1%) patients developed DHF and 6 (9.2%) patients progressed to DSS. None of the patients included in the study expired. The mean duration of hospital stay was 3.7 (±1.2) days. Only nine (13.8%) patients had ALT below 41 U/L at presentation, and mean level of ALT at presentation in the whole study population was 201.4 (±418.46) U/L. Table 1 shows group wise distribution of these parameters.

**Table 1:** Group wise distribution of various parameters ALT, alanine aminotransferase; DF, dengue fever; DHF, dengue hemorrhagic fever; DSS, dengue shock syndrome

	Group 1 (DF)	Group 2 (DHF and DSS)	Total (DF, DHF, and DSS)
Number of patients (n)	18	47	65
Mean age (years)	32.94 (±14.1)	38.36 (±13.73)	36.86 (±13.9)
Hospital stay (days)	3.33 (±0.77)	3.89 (±1.20)	3.7 (±1.20)
Mean ALT at presentation (U/L)	131.67 (±244.48)	228.15 (±467.88)	201.4 (±418.46)

Levels of ALT at presentation in groups 1 and 2 were not normally distributed (kurtosis was 15 and 24.5, respectively, for both groups), and variances of both the groups were also not same as the p value of Levene’s test for homogeneity of variances was 0.524 (>0.05). Therefore, Mann-Whitney U test was applied to compare differences between ALT levels of both groups, and p value was found to be 0.018 (<0.05) and Z was -0.2361. Thus, our null hypothesis was rejected, and it was concluded that there was a statistical difference between ALT levels of both the groups. The effect size was calculated to be 0.087. 

## Discussion

DF is a common viral illness throughout the world [10]. Classical DF carries less mortality and less rate of complications as compared to DHF and DSS, which are more severe forms of illness [11]. Studies have shown that dengue virus affects the reticuloendothelial cells; thus, liver involvement is a well-known feature of dengue virus infection [12,13]. The present study found out that only 13.8% DF patients had normal ALT levels. Studies carried out by Ahmed et al., Kittitrakul et al., and Ashfaq et al. revealed that 38%, 31.7%, and 21.4% of patients had normal ALT levels, respectively [14-16]. The mean ALT level of all the patients was 201.4 U/L in our study. This value was comparable to results obtained by Chhina et al. (218.6 U/L) and Manohar et al. (201.1 U/L) [9,17]. Studies done by Kittitrakul et al. and Srivastava et al. showed mean ALT levels of 152 U/L and 121 U/L, respectively, which were lower than the levels observed in our study [16,18].

Patients with classical DF in our study had significantly lower mean ALT levels than patients with DHF and DSS. No similar studies were done locally in Pakistan; however, studies done by Chhina et al. (India), Kittitrakul et al. (Thailand), Manohar et al. (India), and Srivastava et al. (India) showed similar results [9,16-18].

This study has various limitations, including small sample size, non-probability sampling technique, single center-based experience, and non-inclusion of other markers of hepatic dysfunction. Thus, more large-scale studies are needed to evaluate these findings in detail and see whether other biomarkers of hepatic dysfunction also correlate with dengue severity or not.

## Conclusions

Higher serum levels of ALT at presentation can give a clue about possibility of progression to severe forms of DF (DHF and DSS). Thus, patients with higher ALT at presentation should be prioritized and monitored more rigorously, so that clinical deterioration can be picked at earliest and managed timely.

## References

[1] Stanaway JD, Shepard DS, Undurraga EA (2016). The global burden of dengue: an analysis from the Global Burden of Disease study 2013. Lancet Infect Dis.

[2] Imai N, Dorigatti I, Cauchemez S, Ferguson NM (2015). Estimating dengue transmission intensity from sero-prevalence surveys in multiple countries. PLoS Negl Trop Dis.

[3] Pongpan S, Wisitwong A, Tawichasri C, Patumanond J (2013). Prognostic indicators for dengue infection severity. Int J Clin Pediatr.

[4] Khan MIH, Anwar E, Agha A, Hassanien NSM, Ullah E, Syed IA, Raja A (2013). Factors predicting severe dengue in patients with dengue fever. Mediterr J Hematol Infect Dis.

[5] Ahmed A, Alvi AH, Butt A, Nawaz AA, Hanif A (2014). Assessment of dengue fever severity through liver function tests. J Coll Physicians Surg Pak.

[6] Villar-Centeno LA, Diaz-Quijano FA, Martinez-Vega RA (2008). Biochemical alterations as markers of dengue haemorrhagic fever. Am J Trop Med Hyg.

[7] Huerre MR, Lan NT, Marianneau P (2001). Liver histopathology and biological correlates in five cases of fatal dengue fever in Vietnamese children. Virchows Archive.

[8] Masud F, Butt TK, Ali M. Dengue Expert Advisory Group (DEAG), Dengue GCP guidelines 2012 (2020). Dengue Expert Advisory Group (DEAG), Dengue GCP guidelines 2012. http://www.phc.org.pk/downloads/Dengue%20GCP%20GUIDELINES.pdf.

[9] Chhina RS, Goyal O, Chhina DK, Goyal P, Kumar R, Puri S (2008). Liver function tests in patients with dengue viral infection. Dengue Bull.

[10] Parkash O, Almas A, Jafri SW, Hamid S, Akhtar J, Alishah H (2010). Severity of acute hepatitis and its outcome in patients with dengue fever in a tertiary care hospital, Karachi, Pakistan (South Asia). BMC Gastroenterol.

[11] Chen RF, Yang KD, Wang L, Liu JW, Chiu CC, Chang JT (2007). Different clinical and laboratory manifestations between dengue haemorrhagic fever and dengue fever with bleeding tendency. Trans R Soc Trop Med Hyg.

[12] Nguyen TL, Nguyen TH, Tieu NT (1997). The impact of dengue haemorrhagic fever on liver function. Res Virol.

[13] Mohan B, Patwari AK, Anand VK (2000). Brief report. Hepatic dysfunction in childhood dengue infection. J Trop Pediatr.

[14] Ahmed SI, Khalid MA, Baqai HZ, Ali SF, Ranja ZA (2009). Dengue fever in Northern Pakistan: the hepatic implications. JRMC.

[15] Ashfaq MW, Nadeem M, Khalid MA, Shafiq MM, Ahmad SI (2016). Liver biochemistry: difference between dengue fever and non dengue febrile illnesses. JIMDC.

[16] Kittitrakul C, Silachamroon U, Phumratanaprapin W, Krudsood S, Wilairatana P, Treeprasertsuk S (2015). Liver function tests abnormality and clinical severity of dengue infection in adult patients. J Med Assoc Thai.

[17] Manohar B, Latha NM, Kumar BS, Madhavi K, Sivaramadu K, Dudala SR (2015). Clinical profile and liver function in dengue. NIJP.

[18] Srivastava G, Chhavi N, Goel A (2018). Validation of serum aminotransferases levels to define severe dengue fever in children. Pediatr Gastroenterol Hepatol Nutr.

